# Development of therapies for rare genetic disorders of GPX4: roadmap and opportunities

**DOI:** 10.1186/s13023-021-02048-0

**Published:** 2021-10-23

**Authors:** Dorian M. Cheff, Alysson R. Muotri, Brent R. Stockwell, Edward E. Schmidt, Qitao Ran, Reena V. Kartha, Simon C. Johnson, Plavi Mittal, Elias S. J. Arnér, Kristen M. Wigby, Matthew D. Hall, Sanath Kumar Ramesh

**Affiliations:** 1 National Center for Advancing Translational Sciences, National Institutes of Health, Rockville, MD USA; 2 Division of Biochemistry, Department of Medical Biochemistry and Biophysics, Karolinska Institutet, 171 77 Stockholm, Sweden; 3 Department of Pediatrics, University of California, San Diego, San Diego, CA USA; 4 Department of Cellular and Molecular Medicine, University of California, San Diego, San Diego, CA USA; 5 Department of Biological Sciences, Columbia University, New York, NY USA; 6 Department of Chemistry, Columbia University, New York, NY USA; 7 Department of Microbiology and Immunology, Montana State University, Bozeman, MT USA; 8 Department of Cell Systems and Anatomy, University of Texas Health Science Center, San Antonio, San Antonio, TX USA; 9 Research and Development Service, South Texas Veterans Health Care System, San Antonio, TX USA; 10 Department of Experimental and Clinical Pharmacology, Center for Orphan Drug Research, College of Pharmacy, University of Minnesota, Minneapolis, MN USA; 11 Department of Neurology, University of Washington, Seattle, WA USA; 12 Department of Anesthesiology and Pain Medicine, University of Washington, Seattle, WA USA; 13 Center for Integrative Brain Research, Seattle Children’s Research Institute, Seattle, WA USA; 14 In-Depth Genomics, Bellevue, WA USA; 15 Department of Selenoprotein Research, National Institute of Oncology, Budapest, 1521 Hungary; 16 Department of Pediatrics, Division of Genetics, San Diego and Rady Children’s Hospital-San Diego, University of California, San Diego, CA USA; 17 Rady Children’s Institute for Genomic Medicine, San Diego, CA USA; 18 CureGPX4.org, Seattle, WA USA

**Keywords:** Sedaghatian-type spondylometaphyseal dysplasia, SSMD, Glutathione peroxidase 4, GPX4, Rare genetic disorder, Therapy development, Roadmap, Ultra-rare disease

## Abstract

**Background:**

Extremely rare progressive diseases like Sedaghatian-type Spondylometaphyseal Dysplasia (SSMD) can be neonatally lethal and therefore go undiagnosed or are difficult to treat. Recent sequencing efforts have linked this disease to mutations in *GPX4*, with consequences in the resulting enzyme, glutathione peroxidase 4. This offers potential diagnostic and therapeutic avenues for those suffering from this disease, though the steps toward these treatments is often convoluted, expensive, and time-consuming.

**Main body:**

The CureGPX4 organization was developed to promote awareness of GPX4-related diseases like SSMD, as well as support research that could lead to essential therapeutics for patients. We provide an overview of the 21 published SSMD cases and have compiled additional sequencing data for four previously unpublished individuals to illustrate the genetic component of SSMD, and the role of sequencing data in diagnosis. We outline in detail the steps CureGPX4 has taken to reach milestones of team creation, disease understanding, drug repurposing, and design of future studies.

**Conclusion:**

The primary aim of this review is to provide a roadmap for therapy development for rare, ultra-rare, and difficult to diagnose diseases, as well as increase awareness of the genetic component of SSMD. This work will offer a better understanding of GPx4-related diseases, and help guide researchers, clinicians, and patients interested in other rare diseases find a path towards treatments.

**Supplementary Information:**

The online version contains supplementary material available at 10.1186/s13023-021-02048-0.

## Introduction

Sedaghatian Type Spondylometaphyseal Dysplasia (SSMD) is an ultra-rare, often neonatally lethal, disease first reported by Sedaghatian in 1980 [1]. Since then, 21 individuals are noted in literature who share a characteristic pattern of skeletal anomalies, central nervous system malformations, hypotonia, cardia arrhythmias, and early mortality due to respiratory failure [2–14]. In 2014, Smith and colleagues studied two unrelated families with SSMD using whole exome sequencing and identified bi-allelic truncating variants in the *GPX4* gene, which encodes glutathione peroxidase 4 (GPX4), thus proposing a molecular basis for SSMD [13]. This genetic component of SSMD was further supported in an additional study which used dry blood spots to identify the cause of death for two siblings suffering SSMD symptoms. Fedida et al. identified a novel homozygous GPX4 variant in both siblings [14]. Here, four additional living pediatric patients with different bi-allelic variants in *GPX4* have been identified. These children express typical features of SSMD, but also extend the described disease spectrum to include other phenotypes such as skeletal anomalies, optic nerve hypoplasia, auditory neuropathy, dysphagia, seizures, and profound global developmental delay. As parents of one of the patients, the corresponding author (S.K.R.) and wife started an organization, CureGPX4, to prioritize patient-focused therapies and push the discovery timeline forward [15]. Facing this initial diagnosis, a path forward was not immediately clear, and navigating the steps to a treatment plan is more convoluted with an ultra-rare disease. While much remains to be learned about the clinical spectrum of disease manifestations, we are connecting a growing network of experts to understand the fundamental biology of GPX4. Our goal is to find treatments that improve the quality of life for children with *GPX4*-related disorders within the next 6–12 months, while building a collaborative effort for better understanding the fundamental biology of GPX4.

The goal of CureGPX4 is ambitious. There have been over 7000 rare diseases described, but only ~ 5% of them have at least one treatment approved by the Food and Drug Administration (FDA) [16]. While there are initiatives aimed at speeding up therapeutic development for rare diseases, traditional small molecule drug discovery takes several years to complete, can cost billions of dollars, and identified therapeutic candidates have a low probability of clinical success [17]. Emerging technologies such as gene therapy or antisense oligonucleotides (ASO) have a faster development timeline, but can still be in the order of years, and in some cases are tailored for each patient (n = 1 treatment). In other cases, such as in mitochondrial encoded genes, such technologies have not been developed, even in an experimental setting. In addition to the reducing time, CureGPX4 would need to raise several million dollars, produce relevant scientific discoveries, build a patient community, stimulate biotech industry investment, conduct clinical trials, and secure regulatory approvals to bring therapeutics to patients. Like other rare disease communities, CureGPX4 neither has the money, nor, more critically, do our children have the time to let this process play out.

As a critical first step, we (CureGPX4) have created a new roadmap for therapy development capable of meeting our lofty goal by applying a few guiding principles, namely: seek incremental therapies; prioritize saving time over money; and fail fast to maximize learning. We created the CureGPX4 Roadmap by working backwards from patient needs, aiming for therapies which may first slow, then halt, and finally reverse disease progression. In the first two weeks, we identified eight FDA-approved small-molecule drugs that could have benefits by manually searching the literature. A few of the SSMD patients have begun courses of treatment using these drugs and some have even reported improvements, albeit anecdotally. We will next conduct a longitudinal natural history study, aim to identify reliable biomarkers for disease symptoms, invest in understanding the underlying disease biology, create disease models, and unify all the activities under a novel drug development pipeline, ultimately aiming to identify and validate treatment protocols. The pipeline is open to repurposing existing drugs or drug combinations, novel small-molecules, and drugs based on emerging technologies like gene therapy, ASOs, and gene editing. We aim to test several drugs in multiple preclinical disease models at once to reduce selection bias. We will rapidly make all our results publicly available. This will allow us to leverage the broader scientific community, to identify lead drugs with maximum efficacy and facilitate novel discoveries with regards to disease mechanisms. By approaching the treatment using a network approach, we will break the silos and foster collaboration between our research, industry, and physician partners, and encourage exchange of data and materials.

In this paper, we present our roadmap in greater detail. Typically, rare disease foundations have shared their success stories retroactively as roadmaps [18]. However, such roadmaps lack the high-resolution details and context to help an organization like ours tackle a new rare disease. We thus felt the overwhelming urgency to share our roadmap, however preliminary and optimistic, to help other rare disease organizations in a similar position. By reflecting on the decision-making processes, the structure of our collaborative effort, and the early successes and missteps, we hope to provide insight into the unique challenges of tackling ultrarare diseases.

This article provides an overview of our current understanding of *GPX4*-related disease, which has not been summarized previously. The roadmap was created in collaboration between patient parents and advocates, scientists, and clinicians. It was created based on a newly cemented understanding of the genetic relationship between GPX4 and its clinical manifestations, but with effectively no detailed knowledge of the underlying disease pathogenesis. The roadmap sets forth our suggested translational science principles and logistics that would be needed to enable breakthrough advancements necessary for treatment. The roadmap emerged from a virtual workshop held on March 19, 2020. Because CureGPX4 is a collaborative network and is open to feedback, we appreciate new ideas, help and guidance from the community. We are committed to periodically publishing updates to our roadmap. We hope that by openly sharing this roadmap and materials such as the Investigational New Drug (IND) Template, Roadmap Chart, Conference Guide, among others, we will facilitate other rare disease organizations increase their chances of success (Additional files 1, 2, 3, 4, 5).

### Sedaghatian-type Spondylometaphyseal Dysplasia (SSMD)

Sedaghatian type Spondylometaphyseal Dysplasia (SSMD) is an extremely rare progressive disorder which is characterized by a multi-system presentation, including cupping/flaring of metaphyses, platyspondyly (flattening of the vertebrae), cardiac arrhythmia, and central nervous system (CNS) abnormalities, including hypogenesis of corpus callosum and cerebellar hypoplasia (OMIM #250220) [19]. SSMD was first reported by Sedaghatian (after which the disorder is eponymously named) in 1980, reporting two brothers in Iran who each died within the first week of birth, and finding ‘severe congenital metaphyseal involvement, mild rhizomelic shortness of upper limbs, and mild platyspondyly’. Since that time, a small number of further reports have been published describing patients with presumed SSMD (Table 1).

**Table 1:** Clinical characteristics of published and present SSMD cases

Individual/case	Sedaghatian [1]			Opitz [2]	Campbell [3]		Peeden [4]	Elçioglu [5]		Kerr [6]			Koutouby [7]	Foulds [8]	English [9]	Mehendran [10]	Witters [11]	Aygun [12]	Smith [13]		Fedida [14]		This report			
	1	2	3	4	5	6	7	8	9	10	11	12	13	14	15	16	17	18‡	18‡	19	20	21	22	23	24	25
Sex	M	M	F	M	M	M	M	M	M	M	M	M	M	F	F	M	F	M	M	F	M	F	M	F	M	M
Race	IR	IR	IR	IR	C	C	BA	PA	ME	PA	PA	PA	YE	C	C	C		TU	TU	IR	AM	AM		IQ	IQ	IN
Consanguinity	−	−	−	+	−	−	−	−	−	+	+	+	−	−	−	+	−	+	+	−	+	+		+	+	−
Sibling occurrence	+	+	+	−	+	+	−	−	−	+	+	+	−	−	−	−	−	−	−	−	+	+		+	+	−
Birth weight (kg)		3.3	3.8	3.28			3.12	2.9		2.6	3	2.95	3.4	3.34	2.83	2.54	0.21	3.3	3.3		3.91	1.59		3.4	3.4	
Gestation (weeks)					30	41	40	36		36	38	38	40	38	37	39	18	37	37		39	35		40	40	38
Death (day)	3	3	1	4	SB	1	2	SB	30	SB	1	1	161	30	17	3	TP	120	120	18	4	2	244	NA 6 yr*	NA 11 yr*	NA 2 yr*
Normal intrauterine growth	+	+	+	+		−	−	−	−	−	+	−	+	+	+	+	−				+			+	+	
*Skeletal abnormalities*																										
Short neck			+		+	+	+			−	−	−	+	+	−									−	−	
Rhizomelic shortening of long bones	+	+	+	+	+	+	+	+	+	+	+	+	+	+	+		+	+	+	+			+	−	−	+
Metaphyseal cupping/flaring/irregularity	+	+		+	+	+	+	+	+	+	+	+	+	+	+	+	+	+	+	+	+	−	+	+	+	+
Epiphyseal ossification	+	+		+	+	+	+	+	+	−	−	−		+				+	+					−	−	
Narrow/small chest	−	+		−	−	−	−	+	+	−	−	−	+	−	−		−						+	−	−	
Cupping of rib ends	+	+		+	+	+	+	+	+	−	−	−	+	+	+	+	+				+	−	+	−	−	
Platyspondyly	+	+		−	+	+	+	+	+	+	+	+	+	+	+	+	+	+	+	+				−	−	+
Flared iliac wings	+	+		+	+	+	+	+	+	+	+	+		+		+		+	+	+	+	−	+	+	+	
Lacy/irregular iliac crest	+	+		+	+	+	+	+	+	+	+	+	+					+	+							
Facial abnormalities																										
Telecanthus/hypertelorism										−	−	−	+	−	−			+	+					−	−	
Wide/flat nasal bridge		+	+	+						−	−	−	−	−	−		+							−	−	
Asymmetrical/abnormal ear placement		+	+	+			+			−	+	−	−	+	−									−	−	
Micrognathia				+	+	+							+	+	+			+	+					−	−	
*Nervous system abnormalities*																										
Simplified sulcal/gyral pattern						−	+						+	+	+		+	+	+	+				−	−	
Seizures						−			+				+		+			+	+					−	+	−
Auditory neuropathy																							−	+	−	+
Other				+	+	−	+		+				+	+	+		+	+	+	+				+	+	+
Cardiac abnormalities/arrhythmia																		+	+	+	+		+	+	+	+
Hypotonia		+											+		+			+	+		+	+	+	+	+	+
Delayed development																							+	+	+	+

+ denotes characteristics that are mentioned in each publication, −denotes characteristics that are mentioned as missing in each publication, blank boxes denote no mention of the characteristics. IR, Iranian; C, Caucasian; BA, Black American; PA, Pakistani; ME, Middle Eastern; YE, Yemeni; TU, Turkish; IN, Indian; AM, Arab Muslim; IQ, Iraqi, SB, still birth; TP, terminated pregnancy. ‡, Individual 18 was originally reported in Aygun et al., and additional genetic testing of parents was included in the Smith et al. publication. Note that first authors are described along top of table, cited [1–14]

### The emerging spectrum of GPX4-related disease

**Fig. 1:**
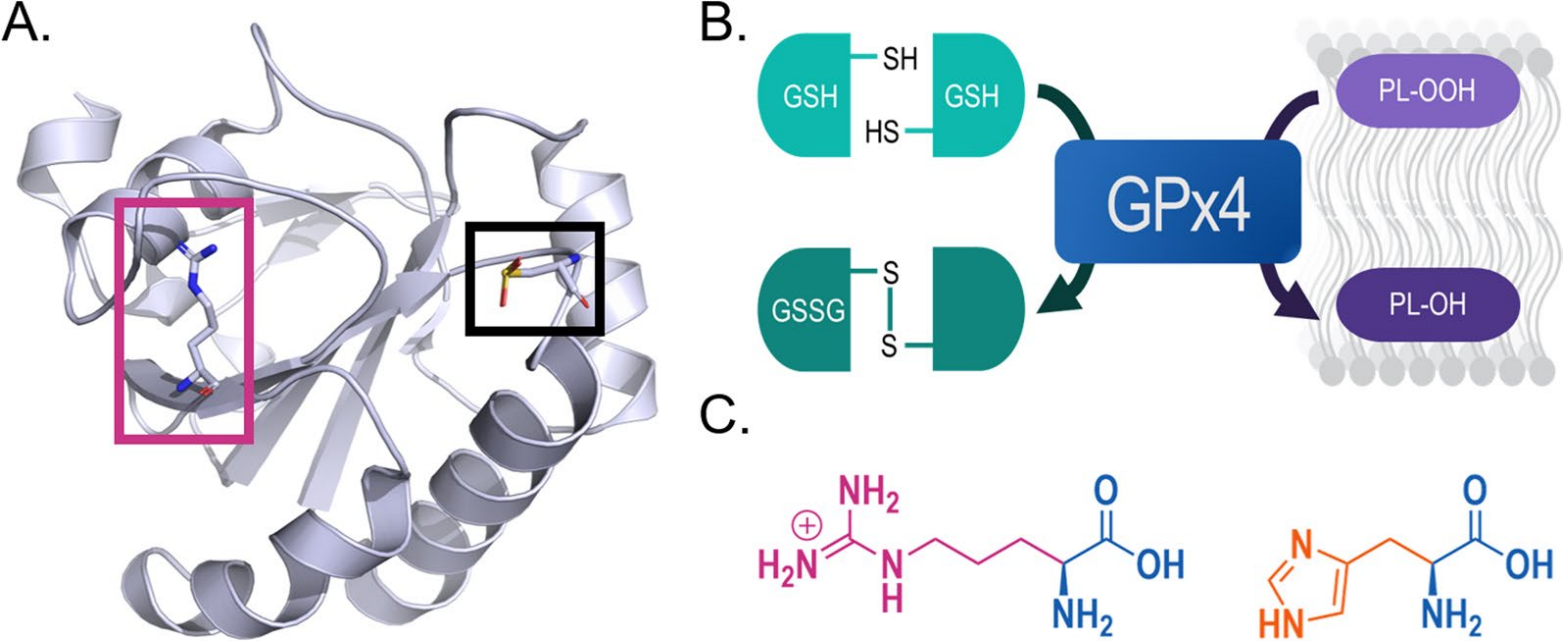
Glutathione peroxidase 4. **a** Crystal structure representation of Sec-containing human GPX4 (Modified from PDB 5H5S) [34]. Black and pink boxes denote selenocysteine (Sec46) and arginine (Arg152) residues, respectively. **b** Schematic of antioxidant function of GPX4. Briefly, GPX4 slows lipid peroxidation by reducing a reactive phospholipid hydroperoxide (PL-OOH) to a non-reactive alcohol (PL-OH) while converting reduced glutathione (GSH) to oxidized glutathione disulfide (GSSG). **c** Comparison of arginine (pink) and histidine (orange) amino acid residues that are substituted in most common R152H GPX4 mutation

At the onset of writing, we know of four pediatric patients (3 male, 1 female, median age 31 months) living with this condition, though sadly one patient has since died. A small number of patients’ GPX4 gene sequences have been reported (Table 2) and include both point mutations, missense mutations, as well as improper splicing. The newly reported cases in this report harbor homozygous point mutations causing a substitution of arginine (Arg, R) at position 176 with histidine (His, H) (Fig. 1). Though studies are ongoing, the implications on GPX4 are discussed below. Based on natural history data of these patients, additional symptoms include severe hypotonia, global development delays, auditory neuropathy, cortical visual impairment, scoliosis, and hypertonia. The oldest patient developed intractable seizures at the age of 3 and continues to be treated with anticonvulsants to reduce the occurrence of breakthrough seizures. Currently, there are no specific treatments for these GPX4-related diseases, except for physical and occupational therapies. Without any treatment, those born with this condition are unable to sit up or walk, have persistent feeding difficulties, and display significantly delayed physical and cognitive development. They are at a high risk for premature death by cardiovascular, cerebrovascular, neuromuscular, or renal complications.

**Table 2:** Summary of molecular genetic findings for select individuals

Ind	Sex	Molecular Effect	Amino acid substitution	GPX4 variant
18	M	NS	p.(Tyr127*)	Not available for affected child
				GPX4(NM_001039848.1):c.381C > A both parents
19	F	FS	Exon 4 splice error	GPX4(NM_001039848.1):c.587 + 5G > A;
		PT	Exon 5 skip	GPX4(NM_001039848.1):c.588-8_588-4del
20	M	FS	p.(His52fs*1)	GPX4(NM_002085.4):c.153_160del
21	F	FS	p.(His52fs*1)	GPX4(NM_002085.4):c.153_160del
22	M	NS	(p.(Gly148Argfs*?));	GPX4(NM_001039848.1):c.441dup maternal;
			(p.(Pro138Arg))	GPX4(NM_001039848.1):c.413C > G paternal
23	F	MS	p.Arg152His	GPX4(NM_001039848.2):c.647G > A;homozygous
24	M	MS	p.Arg152His	GPX4(NM_001039848.2):c.647G > A, homozygous
25	M	MS	p.Arg152His	GPX4(NM_001039848.2):c.647G > A, homozygous

NS, nonsense; MS, missense; FS, frame shift; PT, premature truncation. When available, variants are listed from the individuals in question, and are listed from parents when noted. References included in Table 1

### Role of GPX4 in health and disease

Glutathione peroxidases (GPXs) are a family of selenoprotein antioxidant enzymes that utilize glutathione (GSH) to reduce hydrogen peroxide, and other hydroperoxides, preventing oxidative damage in the cell. Also referred to as Phospholipid Hydroperoxide Glutathione Peroxidase (PHGPX), GPX4 is unique in its monomeric structure and high affinity for lipid hydroperoxides [20, 21]. As a selenoprotein, GPX4 contains the rare amino acid selenocysteine (Sec, U) in its active site (position 73 of 197), often termed the ‘21st amino acid’. The catalytic activity of Sec is indispensable for normal enzyme activity of GPX4 [22] (Fig. 1).

The human GPX4 gene contains seven exons and six introns and can be expressed as three isoforms of the protein, mitochondrial (mGPX4, UniProt P36969-1), cytosolic (cGPX4, UniProt P36969-2) and nuclear (nGPX4) [23]. All three isoforms seem to be ubiquitously expressed in all tissues, and mature mitochondrial and cytoplasmic isoforms are identical following post-translational modifications [24]. The mitochondrial and cytosolic isoforms are known to be essential in somatic cells including neurons of the developing brain [25–28], while the nuclear isoform is predominantly synthesized during late spermatogenesis [29]. Mouse models have shown that the enzyme is important for normal embryogenesis, maintaining mitochondrial oxidative phosphorylation, preventing lipid peroxidation, and playing a part in combating increased oxidative damage due to injury or chemotherapy [22, 30–33].

I. GPX4 prevents lipid peroxidation
  GPX4 can reduce complex lipid peroxides such as those present in lipid membrane bilayer of cells. Polyunsaturated fatty-acid-containing phospholipids (PL-PUFAs) have been shown to be the lipids species most susceptible to peroxidation, with the bis-allylic carbons being most susceptible hydrogen atom abstraction [35, 36]. GPX4 localizes to lipid membranes where it accesses hydrophobic membrane lipids and reduces PL-PUFA hydroperoxides using reduced GSH as electron donor for the reaction [36].
II. Loss of GPX4 can lead to ferroptosis
  Ferroptosis is a distinct form of iron-dependent organized cell death [37–39]. Loss of GPX4 results in higher peroxidation levels of lipids in the cell membrane, triggering ferroptosis. Depletion of the cofactor of GPX4, glutathione, also leads to ferroptosis. Cell death with oxidized levels of phospholipids acylated with polyunsaturated fatty acids, involvement of redox-active iron, and a defective lipid peroxide repair, are the hallmark features of ferroptosis [40]. The antioxidant compound α-Tocopherol (Vitamin E) can stop lipid peroxidation, and thereby slow ferroptosis, as can iron chelators [41]. Also the enzyme recently named ferroptosis-suppressing protein 1 (FSP1) can, in certain cells, act both instead of, and in parallel with, GPX4 to reduce oxidized phospholipids and thereby also suppress ferroptosis [42, 43].
  Ferroptosis has emerged as a mechanism of cell death relevant to multiple diseases including cardiovascular diseases [44], acute kidney failure [45], and CNS disorders [46, 47]. Ferroptosis can, at least in certain cell types, be driven by loss of activity of GPX4, and subsequent accumulation of lipid hydroperoxides. Depletion of GPX4 in mice is known to induce ferroptotic cell death in embryo, testis, brain, liver, heart, and photoreceptor cells [48], cause rapid motor neuron degeneration and paralysis [49], promotes cognitive impairment [50], triggers acute renal failure [51], and results in impaired T-cell-mediated immune response [52]. Mice with depleted GPX4 showed hallmarks of ferroptosis including an increase in lipid peroxidation in various cell types [50].
III. GPX4 maintains mitochondrial phosphorylation
  GPX4 has been shown to protect mitochondrial ATP generation by preventing oxidative damage to mitochondrial structures [28]. Knockdown studies of GPX4 results in a reduction in the expression of genes encoding components of Complex I, IV, and V [53], while overexpression of mitochondrial GPX4 prevents release of the proapoptotic molecule cytochrome C from mitochondria, suggesting a key role as an anti-apoptotic agent in mitochondrial death pathways [26]. Mitochondrial GPX4 protects cardiac contractile function and preserves electron transport chain activities following ischemia/reperfusion [54].
IV. GPX4 mutations cause SSMD-like symptoms
  Smith et al. first established the pathogenic role of three different variants in GPX4 in causing the Sedaghatian-type Spondylometaphyseal Skeletal Dysplasia (SSMD)-like symptoms [13]. The study included whole exome sequencing (WES) of a female child with SSMD, as well as both parents of a diagnosed child, described previously [12]. The identified variants result in a loss-of-function of GPX4 through deletion or duplication resulting in a frameshift and premature truncation of the protein. Recently, additional evidence of the link between GPX4 mutations and SSMD was presented by Fedida, who performed WES on dry blood spot samples of two affected siblings [14]. The homozygous novel GPX4 variant causes premature truncation of GPX4. Of the four patients reported in this paper, the three surviving patients have the same homozygous missense variant, and one harbored a different (missense and duplication) genotype. Importantly, no cases of SSMD have been reported with sequencing data that are not caused by homozygous mutations in GPX4. This highlights a major need in sample collection at birth to enable proper diagnosis for diseases with high neonatal mortality, as well as emphasizes the important role of WES in disease diagnosis and study.

### Establishing the CureGPX4 organization

The CureGPX4 organization was created with lofty short- and long-term goals by the parents of a patient with SSMD. In the short-term, improvement of quality of life is paramount; to improve mobility, increase independence, and minimize detrimental symptoms. In the long term (3–5 years), we want to develop treatments to address the underlying disease pathology. As the founders lack formal biomedical research training, they have relied on input from other patient groups, academic researchers, and physicians, to establish a structure and course for the organization. Through these efforts, we have created a team of researchers, set clear directions, removed impediments to collaboration, and created a roadmap towards reaching the goal.

**Fig. 2:**
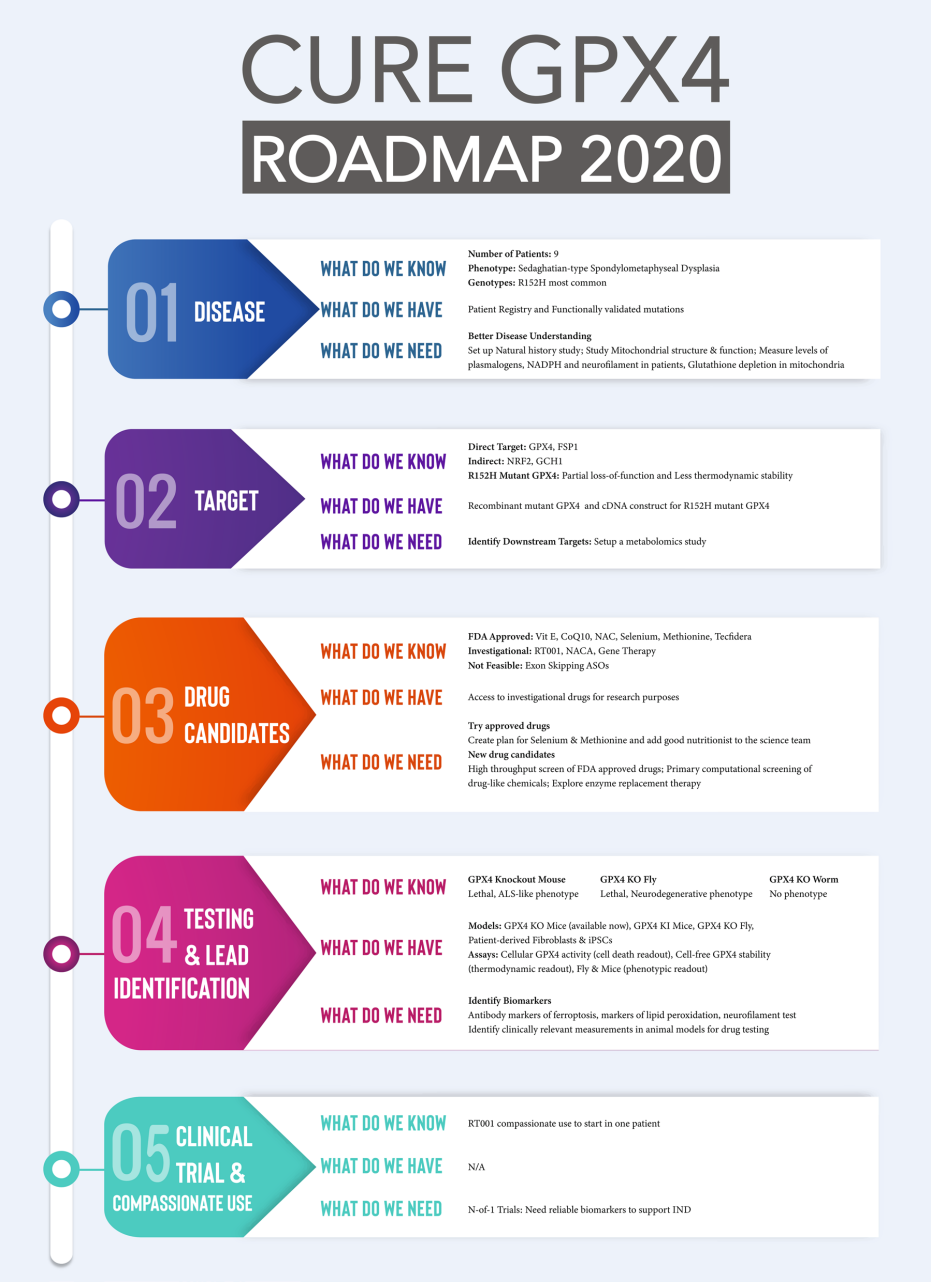
GPX4 Roadmap 2020. Roadmap for GPX4-related disease therapy development

I. Scientific team
The CureGPX4 Team is a cross-disciplinary group of highly collaborative experts sharing the common goal of identifying and developing treatments for GPX4-related diseases broadly, and SSMD specifically [55]. Expertise ranges from physicians to basic and translational scientists, and together we have the structural, functional, drug-development, modeling, and clinical knowhow to advise on a therapy development pipeline (described later in the paper). Starting with patients, parents, and immediate physicians, the team was expanded largely through word-of-mouth in the GPx4 and rare disease-focused communities.
II. Guiding principles
Like other patient organizations, we are focused on finding treatments. But our choice of guiding principles is what dictates the activities we prioritize to find those treatments. The peculiarity in CureGPX4’s roadmap is a direct result of our dedication to the following principles:
  Incremental over big bang—Our initial investments are focused on identifying reasonably efficacious repurposed drugs in a timelier manner rather than pouring financial resources and several years into developing a single, potentially highly-effective drug. This affords us successes in each step: minimizing symptoms, halting disease progression, and eventually improving outcomes.
  Treatment over intellectual property—Our primary focus is not patentable novel technologies or molecules. Rather, we are pushing for repurposing approved drugs, using naturally occurring substances, utilizing existing technology, or adapting generics.
  N-of-1—As our community of patients is extremely small, clinical trials are not practical. Expanded access, commonly called compassionate use, of existing and experimental drugs is our primary avenue for novel treatments [56].
  Emphasize timeliness over costliness—For disease progression as rapid as our patients’ experience, time is the most limited resource. Therefore, we are choosing to prioritize speed over financial cost. It does not necessarily mean our organization spends more money. On the contrary, activities that are quick to execute tend to be small and cheap. By choosing to reduce time over money, we not only hope to find treatment faster but also potentially cheaper.
  Embrace early failures—All rare diseases exist in emerging fields of study, SSMD and other GPX4-related diseases included. Instead of trying to prevent failures, we assume failures are inevitable in every activity. We chose to fail fast, fail cheap, maximize the learning from failures, and fail often enough until we learn to do it right.
III. Collaborative Network
Our Scientific Team members are geographically distributed around the world, work at different institutions, are motivated by different goals, speak different languages, and several were unaware of each other until brought together by CureGPX4. To find a treatment, however, a team must collaborate with trust, integrity, shared goals, and a sense of urgency.
  We, therefore, created the CureGPX4 Collaboration Network; a safe and trusted space for the Scientific Team to collaborate. We are in the process of signing Confidentiality Disclosure Agreements (CDAs) and provided templates for team status reports (Additional file 5) with all institutions in the network to facilitate free exchange of ideas, information, results, and protocols without the reservations linked to potential intellectual property. Institutions participating in the network use the standard Uniform Biological Materials Transfer Agreement (UBMTA) template to freely exchange reagents, cells, biological samples, and other materials with each other for the purpose of finding a treatment.
IV. CureGPX4 Research Conference
The *CureGPX4 Research Conference* was held on March 19, 2020. This one-day virtual conference aimed at bringing a diverse group of researchers from the scientific team, clinicians, and industry partners who are working on finding a treatment for SSMD. Additional file 2 contains all the materials used to run the conference including format, agenda, invitations. The primary goal of the conference was to create a Roadmap for Therapy Development by the end of the meeting. The Roadmap for a rare disease should identify experiments necessary to understand the disease, identify drug repurposing opportunities, and explore the use of emerging technologies like gene therapy, ASOs, CRISPR/Cas9 and others to treat this disease.
  The conference was structured with the goal of making decisions to build the roadmap, in addition to sharing information. With 30 participants over 8 h of meeting, we made 20+ decisions to build the roadmap. Figure 2 presents the roadmap chart created at the meeting. The following section explains the roadmap in more detail.

### Roadmap for therapy development

Five critical areas were identified for SSMD: the disease, the target, drug candidates (repurposing and new), testing and lead identification, and clinical trials and compassionate use. (Fig. 2) For each of these areas, three points were addressed that provide clarity and clearly define next steps for CureGPX4: *What do we know? What do we have? What do we need?*

I. Identify patient needs—Status: Complete
To provide a clear vision to the team, the patient population was contacted directly. With only four known patients at the time, CureGPX4 could have face-to-face conversations with each family to understand their experience and the needs of the patients. For a larger patient community, one might need to use scalable tools such as surveys [57].
  Results:
  We sought to identify what patient families expect from the therapies, acceptable tradeoffs between treatment benefit and risk outcomes, and more broadly their dream for relief from the suffering of SSMD. Patient families expressed the following:
  Long-term goal is independence–Functional independence and personal autonomy are key outcomes with the greatest impact for improving quality of life
  Incremental therapies—First slow, then halt, disease progression, then work to reverse symptoms
  Sustainable therapies—Lifelong diseases need lifelong treatments, therefore the therapies we identify must be available to patients for as many years as they need, and have a minimized financial burden
II. Low-throughput drug repurposing—Status: Complete
  There are currently no approved or experimental therapies for SSMD, and the timelines for even well-funded biotech industry research are not ideal for conditions with such severe phenotypes. Guided by the immediate needs of our patients for incremental therapies, the primary focus is to identify existing FDA-approved drugs that can be repurposed for this condition (Table 3).

**Table 3:** Short-listed repurposed treatments for SSMD and GPX4-related diseases

Name	Rationale	Availability	SSMD Status
Vitamin E	Potent antioxidant known to prevent ferroptosis	Over-the-counter	Administered to 2 of 4 patients Dosage: 15 mg 2X/day
N-Acetyl-Cysteine (NAC)	Increases glutathione biosynthesis to boost residual GPX4 activity	Over-the-counter	Administered to 2 of 4 patients Dosage: 300 mg 3X/day
CoQ10	Essential for repair of peroxidized lipids. Acts as an antioxidant	Over-the-counter	Administered to 2 of 4 patients Dosage: 50 mg 2X/day
Selenium	Limiting step in GPX4 production, may increase selenoprotein expression	Over-the-counter	Administered to 1 of 4 patients Dosage: 75 µg 1X/day
L-methionine	Increases glutathione biosynthesis through transsulferation pathway to boost residual GPX4 activity	Over-the-counter	Pending administration
RT001	Protects lipid membranes against peroxidation	In clinical trials for treating multiple indications	Administered to 2 of 4 patients under Expanded Access
Dimethyl fumarate	Activates Nrf2 gene, master regulator oxidative stress response	Approved in USA for treating multiple sclerosis	Evaluating for of off-label use

Collection of potential and in-use repurposed treatments for current SSMD patients
  An initial inquiry into a commercial high-throughput drug screen of 4000 FDA approved molecules on cells or using animal models was quoted to take 9–15 months, cost over $150,000, and would require the development and validation of disease models. The CureGPX4 network is working to develop disease models in fruit fly (*D. melanogaster*), worms (*C. elegans*), and zebrafish (*D. rerio*), validating their phenotypes and testing drugs like the pipeline described by Iyer and colleagues [58]. However, due to small number of identified patients, our understanding of the natural history of the disease limits our ability to design and interpret such screens, making the investment risky.
  To move forward with drug discovery while we advance our understanding of the basic science of SSMD and GPX4-related diseases, a literature review identified FDA approved drugs and supplements predicted to help compensate for the impaired GPX4 function. The search criteria focused on potential treatments that could have one or more of the following effects or drug categories:
  Increase GPX4 protein levels and/or increase residual GPX4 activity
  Increase the activity of GPX4 antioxidant pathways by modifying the activity/expression of other related antioxidant enzymes
  Increase the activity of alternate or compensatory pathways
  Reduce or scavenge the phospholipid oxidation damage resulting from reduced GPX4 activity (e.g., use of antioxidants)
  Drugs that have been found to be effective in similar conditions
    Results:
    Using this approach, we identified 36 FDA approved treatments with reasonable rationales. Of the results, the following compounds were shortlisted based on efficacy and safety. (Expanded list available in Additional file 4)
III. Patient registry and longitudinal natural history study—Status: In progress
  Natural history is a scientific and systematic study of the patients to understand clinical, biological, and social aspects of the disease. Qualitative and quantitative data from natural history studies is critical to understand the course of a disease and its impact on patients and informs the design of clinical trials for therapy development. Natural history studies help physicians recommend disease management strategies, identify unrecognized impacts of disease, give a voice to patients, and ensure regulators can perform an unbiased assessment of trial outcomes. Natural history data can also inform new hypotheses for translational science. For example, many patients with SSMD have optic nerve abnormalities, which could lead to new research into the role of GPX4 in development of optic nerves and vision.
  Prior to collecting natural history data, a patient registry must be established. A registry is simply an up-to-date address book of every patient in a disease population, managed (in this case) by CureGPX4. To collect natural history data, we will design, create and send surveys to every patient in the registry periodically. A qualified individual must create a study protocol and get it approved by an Institutional Review Board (IRB) before starting the study. IRB approval is necessary to collect, store and act on data from human subjects. Surveys are created and sent using standard off-the-shelf software (Sanford CoRDS [59], NORD Registry [60]) or created with a HIPAA compliant software such as RedCap [61–63]. SSMD disease displays rapid progression in the first years of life, so CureGPX4 has decided to send out monthly surveys. We will use a custom RedCap installation to store the data in a compliant manner and retain complete ownership of the data. We will follow FDA’s guidance on natural history study design to collect data in a compliant, useful and stay relevant to drug development in the future [64].
  The visibility of CureGPX4 as a Foundation is essential to ensure that clinicians and patient advocates can connect to. CureGPX4 has established a stand-alone web-page (cureGPX4.org) and also worked with the National Center for Translational Science to create a SSMD page at the Genetic and Rare Disease (GARD) Information Center [65].
IV. Understanding disease biology—Status: In progress
  *“How do variations in GPX4 gene cause SSMD disease?"* Answers to this question will help us identify one or more components of the biological pathway that could be targeted with a drug. Answering this question relies on an understanding of the function of GPX4, and the mechanistic cellular consequences of a total or partial loss of GPX4 function. Based on our current understanding of GPX4, oxidative stress response pathways, and the phenotype of SSMD disease, we have arrived at an initial set of primary research questions:
  *How prevalent are GPX4 mutations, and are the variants pathogenic?*
    To validate the pathogenicity of *GPX4* variants, we will analyze patient-derived fibroblasts for hallmarks of oxidative stress and ferroptosis. We will try to restore the wild-type cellular phenotype by transfecting the cells with wild-type *GPX4* gene, over-expressing wild-type GPX4 protein, and silencing the mutant protein. We will also assess publicly available human genome sequences to study the range and extent of disease-causing and as-yet unknown GPX4 mutations in the human genome
    Opportunities:
    Documented and validated variants leading to SSMD-like characteristics can allow clinical genetic testing companies to label these variants as pathogenic in their reports. This would enable physicians to confidently diagnose patients with this disease
    De-risks other basic science and translational activities that assume pathogenicity of the gene variants
    Peer-reviewed publications on the validated variants and disease will raise awareness of SSMD
*How do variations impact protein structure and function?*
    Variants in the coding region of the gene can change the protein structure. Altered protein structure can lead to total, partial, or no loss (or gain) of function. In some cases, the mutant protein will be catalytically active but less stable within the cell. We will understand the protein’s structure, localization, and expression levels by creating and isolating a recombinant protein with specific variants. We will analyze thermodynamic stability and antioxidant activity using cell-free assays on recombinant protein. We will use computational modelling to predict the protein structure and validate it with X-Ray crystallography.
    To measure cellular activity of protein, we will use reference cell lines with disease-causing GPX4 mutations to look for hallmarks of oxidative stress and ferroptosis, and the rescue of these phenotypes with expression of wildtype protein. We will repeat the assays on patient-derived fibroblasts to get high confidence that the variant is indeed causing the functional changes and nothing else.
    Opportunities
    Unlocks new therapeutic opportunities depending on the nature of the change in protein’s function
    Cell-free assays using recombinant protein will allow us to screen and discover drugs capable of binding to the protein to modulate its activity
    In-vitro assays on fibroblast cells allow us to screen thousands of FDA approved drugs in a high-throughput fashion to identify drugs that could potentially restore cellular function
*How do variations impact cellular structure and function?*
    When observing cellular changes, we want to understand if and how there is a difference between “acute” versus “chronic” oxidative stress condition. A patient with mutated GPX4 since embryonic development could be under “chronic” oxidative stress whereas the oxidative stress in an in-vitro or in-vivo assay knocking down GPX4 could be considered “acute.”
    Cells adapt to the change in gene function by upregulating other pathways. GPX4 uses the cofactor glutathione to scavenge reactive oxygen species (ROS) in the cell membrane, cytosolic and mitochondrial compartments. Loss of GPX4 activity might activate other compensatory genes or pathways in response to increased ROS, such as FSP1 [42, 43]. We will use RNASeq to look at gene expression changes and metabolomics and lipidomics analyses to examine changes in pathways, networks, cellular lipids, and other metabolites. One isoform of GPX4 is trafficked to the mitochondria, and GPX4 has been shown to be critical for mitochondrial function [66], so mitochondrial activity in patient-derived fibroblasts will also be examined.
    Opportunities
    Understanding the cellular consequences of GPX4 disfunction will illuminate novel therapeutic targets and druggable pathways
    Understanding the function of mitochondria in the disease, or any structure or functional changes will open the door for mitochondria-specific therapeutics already available in the market
    A measurable effect of mitochondrial dysfunction in blood or urine samples could open the possibility to identify clinically significant biomarkers of disease
*How do variations impact neurological structure and function?*
    SSMD disease causes developmental delays, and changes in brain structure as revealed through patient MRIs. We will advocate for the study of neurological changes using conditional complete GPX4 knockout mice, GPX4 mutant transgenic mice, and by differentiating patient-derived iPSC lines into neurons.
    Opportunities
    iPSC-derived neurons and brain organoids could be valuable models for drug screening [67]
    Identifying similarities to other neurological conditions will allow better drug repurposing
    Gain greater insight into the impact of ROS regulation on normal neuronal cell function
    Insight into the impact of GPX4 mutations during neurodevelopment [68]
*How do variations impact metaphyseal bone development?*
    Patients with SSMD disease are born with skeletal changes that progresses with age. To our knowledge, there has been no prior work to characterize skeletal morphology in model organisms. We will study the skeletal changes using conditional complete GPX4 knockout mice and GPX4 mutant transgenic mice. We will dive deep into the development of bones and chondrocytes by differentiating patient-derived iPS cells.
    Opportunities:
    Insights on the impact of oxidative stress on bone development could lead to fundamental understanding of biological processes
    Understanding the skeletal progression could open the possibility of using patient’s bone X-rays as one of the endpoints for clinical trials in the future
V. Disease models—Status: In progress
  The purpose of a disease model is to predict a drug’s impact on the quality of a patient's life without giving it to humans. Models should be developed to accurately recapitulate the human disease within the biological system or process they represent ex: biochemical, cellular, whole organism etc. We also want models to be sensitive enough to show a measurable difference when intervened with a drug. In the context of SSMD, ensuring that scientists have identified and can agree on the appropriate ortholog to human GPX4 for manipulation is critical, and as a selenoprotein GPX4 presents further challenges across other species. For example, drosophila and worm (C. elegans) do not express a selenocysteine-containing ortholog of GPX4, and zebrafish appear to have two selenocysteine-containing orthologs of GPX4. On the other hand, mice (and other mammals) have a single selenocysteine-containing ortholog of GPX4.
  With a goal of covering multiple biological systems to study effects on oxidation, the following models have been considered worth pursuing:
  Wild-type and mutant human GPX4 recombinant protein (available now at Karolinska Institute)
  CRISPR-edited GPX4 variant in reference cell lines (available now at Colombia University)
  SSMD patient-derived fibroblasts (available now at RUCDR Biorepository)
  SSMD patient-derived iPSC lines (available now at RUCDR Biorepository)
  Brain organoids derived from patient-derived iPSCs (not yet started)
  GPX4 conditional/complete knock-out mice (available now at JAX, Stock #027964)
  GPX4 condition knock-in mice (in-progress, ETA 1-Feb-2021)
  For genetic conditions, animal models are built by recreating the genetic variation in the animal’s genome or silencing the gene entirely. These are good approximations of the human condition but seldom sufficient to predict the clinical outcome of a drug. Some might argue that patient derived cells, fibroblasts or iPSCs are good predictors of clinical outcome. This might be true in hindsight, but there is no way to determine the ideal model a priori. Based on this observation, we will use multiple models to evaluate a drug to get higher confidence.
VI. Emerging technologies—Status: In progress
  Antisense Oligonucleotides, Gene Replacement Therapy (GRT), and CRISPR-Cas9 gene editing and others have the potential to precisely correct the genetic defects found in our patients. ASOs are designed to skip the exon where mutations occur, in the hopes of restoring the protein’s function albeit partially. We have analyzed wild-type and exon skip GPX4 protein structure in-silico and found destabilization, ruling out ASOs as a possible therapeutic candidate for SSMD disease.
  Gene Replacement Therapies are attractive, especially to deliver a functional copy of GPX4 to neurons that are most susceptible to loss of a key antioxidant. At 2.8 kilobases, GPX4 fits within Adeno-associated virus 9 (AAV9), one of the most common AAV serotypes used in neurological diseases. A hefty investment of $5-7 million and several years’ time could lead to the technological advancements needed to make these treatments a reality.

## Conclusions

Ultimately, CureGPX4 aims to raise awareness of SSMD and the molecular basis that links this disease to GPX4. While the function and pharmacologic disruption of wildtype GPX4 have been explored for a range of neurodegenerative diseases and various cancers, our belief is that all new knowledge in these fields can, and will, assist the diagnosis and treatment for future sufferers. Through our scientific and clinical network, we aim to ensure that a translational science approach to understanding GPX4 biology will lead to new therapeutics for patients suffering from this disease. To this end, we present a framework for a systematic, rapid, and collaborative effort towards therapeutic discovery for SSMD, and any other ultrarare diseases in need.

## Supplementary Information

**Additional file 1**: IND template for compassionate use.

**Additional file 2**: Conference format and guide,

**Additional file 3**: Roadmap chart template.

**Additional file 4**: Manual drug repurposing chart.

**Additional file 5**: Weekly status reports template

## Data Availability

The datasets and templates supporting the conclusions of this article are included within the article and its Additional files 1, 2, 3, 4, 5. Additional information may be available from the corresponding author on reasonable request.

## Abbreviations

AAV: Adeno-associated virus
Arg: Arginine
ASO: Allele-specific oligonucleotide
ATP: Adenosine triphosphate
CoQ10: Coenzyme Q10
CDA: Confidential disclosure agreement
cGPX4: Cytoplasmic GPX4
CNS: Central nervous system
FDA: U.S. food and drug association
FSP1: Ferroptosis suppressor protein 1
GCH1: GTP cyclohydrolase 1
Gly: Glycine
GPX4: Glutathione peroxidase 4
GRT: Gene replacement therapy
GSH: Glutathione reduced
GSSG: Glutathione disulfide, oxidized
His: Histidine
IND: Investigational new drug
iPSC: Induced pluripotent stem cell
IRB: Institutional review board
mGPX4: Mitochondrial GPX4
NAC: N-acetylcysteine
NACA: N-acetylcysteine amide
Nrf2: Nuclear factor erythroid 2-related factor 2
nGPX4: Nuclear GPX4
PHGPX: Phospholipid hydroperoxide glutathione peroxidase 4
PL: Phospholipid
PL-OH: Phospholipid alcohol
PL-OOH: Phospholipid hydroperoxide
PL-PUFA: Phospholipid containing n-3 polyunsaturated fatty acids
Pro: Proline
ROS: Reactive oxygen species
Sec: Selenocysteine
SSMD: Sedaghatian type spondylometaphyseal dysplasia
Tyr: Tyrosine
Vit E: Vitamin E
WES: Whole exome sequencing
