# Supplementary material for: Development of therapies for rare genetic disorders of GPX4: roadmap and opportunities
Source: Orphanet J Rare Dis. 2021 Oct 23;16:446. doi: 10.1186/s13023-021-02048-0 (PMC8542321; doi:10.1186/s13023-021-02048-0)
Supplement: Supplementary file 1 — Additional file 1: IND template for compassionate use. [file 13023_2021_2048_MOESM1_ESM.pdf]

6-January-2020

Food and Drug Administration
Center for Drug Evaluation and Research
Central Document Room
ATTN: Division of Gastroenterology and Inborn Errors Products (DGIEP)
"EXPANDED ACCESS SUBMISSION"
5901-B Ammendale Rd.
Beltsville, Md. 20705-1266
(301) 796-2120

**Re:**	Original Sponsor-Investigator IND Application –<br>***Expanded Access Request for a Single Patient per 21 CFR §312.310(b)***
**Drug Product:**	EXAMPLE-001
**Sponsor-Investigator:**	Dr. John Doe, MD, PhD
**Commercial Sponsor:**	Example, Inc.
**Associated IND:**	<IND Number provided by the company>
**Clinical Studies:**	<Clinical study number provided by the company>

Dear Sir or Madam,

Enclosed, please find an original, and two exact electronic copies of an original Sponsor-Investigator IND (IND). This is an expanded access request for a single patient per 21 CFR §312.310(b).

In this IND, we request use of EXAMPLE-001 drug for a 2 year old male, with Spondylometaphyseal Dysplasia Sedaghatian Type (SSMD) patient. SSMD is an autosomal recessive rare genetic disease resulting from deficiency of *Glutathione Peroxidase-4* (*GPX-4*) gene. It is a neonatal lethal form of spondylometaphyseal dysplasia characterized by severe metaphyseal cupping, platyspondyly, cardiac arrhythmia, brachydactyly, and central nervous system abnormalities. The majority of patients die in the first days of life with symptoms of cardiorespiratory insufficiency. Loss-of-function mutations in the GPX4 gene are known to cause this condition. However the patient has survived his first year of life with developmental delay.

Glutathione Peroxidase-4 is a lipid repair enzyme produced by the GPX4 gene. It is known to reduce lipid peroxidation by scavenging reactive oxygen species using Glutathione as the substrate. Loss of GPX4 function has been shown to increase lipid peroxidation triggering cell death through ferroptosis. Cells with ablation of GPX4, in-vitro, have been rescued by EXAMPLE-001 by halting the lipid peroxidation chain reaction.

EXAMPLE-001 drug is a site-specific synthetic homologue of naturally occurring citric acid. It has been established that substitution of phosphorus with hydrogen at bis-allylic sites, as in EXAMPLE-001, decreases the production of lipid peroxidation products.

Given these results, we believe EXAMPLE-001 could help slow down or stop disease's progression. Without intervention, the natural progression of his disease will result in significant impairment in his day-to-day functioning with a high risk to survival. EXAMPLE-001 is safe, well-tolerated in the pediatric population, without any serious adverse events reported in previous studies (see Section 1.3).

Background information on SSMD and the effects of using EXAMPLE-001 can be found in Section 1 of this IND. The proposed treatment protocol for the patient is described in Section 2. Preclinical testing of EXAMPLE-001 model of disease can be found in Section 1.3. The reference rights to the IND for EXAMPLE-001 from Example Inc, the drug manufacturer, can be found in Attachment 1, along with a letter of Authorization for compassionate use.

We thank you in advance for your consideration of our IND submission to treat this rare disease. If you have any questions or need any additional information, please do not hesitate to contact Dr. John Doe by phone 111-111-1111 or by e-mail at john.doe@example.org.

Regards,

Dr. John Doe, M.D., Ph.D.,

1 Example Way,

Seattle, Washington, 90001

United States

Sponsor-Investigator Investigational New Drug (IND) Application

Original Submission – Expanded Access Request for a Single Patient per 21 CFR §312.310(b)

FOR

EXAMPLE-001 in a Single Patient with Spondylometaphyseal Dysplasia Sedaghatian Type

6-January-2020

Sponsor-Investigator

Dr. John Doe, M.D., Ph.D.,

1 Example Way,

Seattle, Washington, 90001

United States

Drug Manufacturer

Example, Inc.

1 Example Way,

Los Angeles, CA 90001

Phone: 111-111-1111

Abbreviations

AE	Adverse Event
PUFA	Polyunsaturated Fatty Acid
D-PUFA	Deuterated Polyunsatured Fatty Acid
SSMD	Spondylo-Metaphyseal Dysplasia -Sedaghatian Type

Table of Contents

Abbreviations 4

1 Background 7

1.1 Disease 7

1.1.1 Natural History 7

1.1.2 Loss of GPX4 function causes disease 7

1.2 The Patient – John Doe 7

1.3 Drug - EXAMPLE-001 8

2 Proposed Treatment Plan 9

2.1 Dosing Scheme 9

2.2 Criteria for Discontinuation of the Drug 9

2.3 Risk/Benefit 10

3 Chemistry Manufacturing and Controls 11

4 Pharmacology and Toxicology Information 11

5 Previous Human Experience 11

6 References 11

Attachment 1: Example Inc's Reference Rights Letter and Letter of Authorization 12

Attachment 2: John Doe – Medical History 13

Attachment 3: Clinical Presentation of other patients with p.R216H mutation 14

Attachment 4: Proposed Treatment Protocol 15

Attachment 5: Draft Informed Consent Form (ICF) 16

Attachment 6: Example Inc's Investigational Brochure and Rationale to Treat SSMD 17

Attachment 7: CV – Dr. John Doe, MD, Ph.D. 18

1 Background

1.1 Disease

Spondylometaphyseal dysplasia Sedaghatian type (SSMD) is a neonatal lethal form of spondylometaphyseal dysplasia characterized by severe metaphyseal cupping/flaring, mild shortness of the upper limbs, and mild platyspondyly. A lacy appearance of the iliac crests, cardiac arrhythmia, a narrow chest, brachydactyly, and central nervous system abnormalities including hypogenesis of the corpus callosum, cerebellar hypoplasia have been described. The majority of patients die in the first days of life with symptoms of cardiorespiratory insufficiency. Only nine cases have been reported so far.

1.1.1 Natural History

Majority of patients die in the first days of life. Our understanding of the disease is based on clinical reports or autopsy findings. There is no data around the progression and natural history of the disease.

1.1.2 Loss of GPX4 function causes disease

Smith et al., 2014 established the pathogenic role of mutated *GPX4* in this disease and reported three variants. In addition to the three pathogenic variants, ClinVar database reports one variant of uncertain clinical significance, making it a total of four clinically relevant variants in *GPX4*.

The selenoprotein GPX4 is an antioxidant defense enzyme that protects cells against membrane lipid peroxidation. GPX4 uses reduced Glutathione to convert lipid hydroperoxides to lipid alcohol and prevents the iron-dependent formation of lipid reactive oxygen species. Inhibition of GPX4 leads to lipid peroxidation and results in a non-apoptotic cell death called ferroptosis.

All the four reported variants result in a loss-of-function of GPX4 through deletion or duplication resulting in a frameshift and premature truncation of the protein. We believe SSMD is caused by reduction in GPX4 function leading to ferroptosis across cardiac, skeletal and nervous systems.

1.2 The Patient

Our patient, John Doe, is a 2 year old male born at 40 weeks' gestation with intrauterine growth restriction and Apgar scores 8 and 8 at one and five minutes respectively. He was born with microcephaly, hypotonia, stridor, optic nerve hypoplasia, sensorineural hearing loss, and feeding difficulties. He currently receives nutrition through a G-tube, missed all major developmental milestones, lacks head control, and cannot sit unassisted. At 2 years of age, his abilities are equivalent to a 2 month old baby for fine and gross motor skills, 5 month old for

cognitive skills, and 9 months of age for social-emotional skills. He is at less than 1 percentile for weight and height with short stature.

Bone surveys performed 45 days after birth show findings consistent with cupping of the metaphyses and normal epiphyses. He had zones of provisional calcification of the metaphysis throughout and a lacy ilium. There was no evidence of platyspondyly at this time. However, cervical spine film performed at 9 months of age showed considerable platyspondyly throughout the cervical spine with hypoplasia of T12, but no evidence of subluxation or instability. This is consistent with the skeletal presentations of SSMD.

In contrast to known features of SSMD, John's brain MRI performed at 30 days of age and repeated at 1.5 year of age showed interval cerebellar atrophy and supratentorial white matter atrophy. No seizure activity was detected in EEG. No cardiac arrhythmia was detected by Holter monitoring. He had an unrevealing endocrinologic work-up, metabolic panel, creatine kinase, acylcarnitine profile, carbohydrate deficient transferrin and no known renal anomalies. Selenium levels and GSH-GSSG ratio in whole blood samples were within normal range.

Whole exome sequencing identified a homozygous missense variant of uncertain significance in *GPX4* gene (c.647G>A, p.R216H) inherited recessively. Both parents are heterozygous carriers of the same variant. We were not able to confirm that the homozygous variant is the cause of John's features as there are only 2 cases reported to our knowledge which have linked the *GPX4* gene with clinical diagnosis of SSMD. However, John's clinical features do fit with the clinical diagnosis of this condition.

In *Attachment 3 (Clinical Presentation of other patients with p.R216H mutation)*, we show the clinical presentation of two other patients with p.R216H mutation. John Doe and the two patients belong to different ancestry yet have striking similarities in their clinical presentation of the condition. This furthers the support to establish the pathogenic role of the homozygous variant. The partial loss-of-function is also consistent with John's milder phenotype and longer survival compared to other patients.

1.3 Drug - EXAMPLE-001

Detailed information about the drug, mechanism of action, pre-clinical and clinical data is available in *Attachment 6 (Example Inc's Investigational Brochure and Rationale to Treat SSMD)*. Here is a summary:

<Summary of EXAMPLE-001 drug; mechanism of action; how it is known to help with the disease; Previous pre-clinical uses/experiences of EXAMPLE-001 with a highlight on safety profile>

2 Proposed Treatment Plan

This is a single patient, compassionate use trial of EXAMPLE-001. The primary objective is to slow down the progression of John Doe's symptoms. A secondary objective is to evaluate the safety, tolerability and feasibility of administration of EXAMPLE-001 in a single subject with Spondylometaphyseal dysplasia Sedaghatian type (SSMD).

The full treatment protocol can be found in *Attachment 4 (Proposed Treatment Protocol)* of this submission. The schedule of assessments is provided in Table 1 of the protocol. A draft informed consent form is provided in *Attachment 5 (Draft Informed Consent (ICF))*. Following is a brief description of the dosing scheme and some of the key additional safety measures in place for John Doe.

2.1 Dosing Scheme

Based on previous studies, the recommended dose suggested for John is 2 capsules BID upto a total dose of 4 capsules. If John Doe is unable to tolerate study drug because of adverse events, the dosing schedule may be changed at the discretion of the investigator. The total dose may be given TID or may be reduced by 1-2 capsules/day as needed.

2.2 Criteria for Discontinuation of the Drug

EXAMPLE-001 will be continued for period of 1 year, unless one or more of the following occurs:

- There is a life threatening adverse event (AE) related or possibly related to treatment with EXAMPLE-001
- There is significant deterioration of John Doe's overall health status due to progression of SSMD
- If both of John Doe's parents decide to stop treatment at any time, for any reason
- If we identify any potential new side effects or markers related to the EXAMPLE-001, then we will discontinue

The safety assessments for the drug include physical, neurologic and orthopedics examinations, vital signs, 12-lead ECG and clinical laboratory tests (hematology, clinical chemistry, lipid profile, and coagulation) to identify adverse events (AEs). Adverse events will be evaluated for incidence, severity, and relationship to study drug.

2.3 Risk/Benefit

SSMD is caused by a loss of GPX4 protein function leading to cell death through a process called Ferroptosis. Ferroptosis is emerging as a mechanism of cell death in various diseases including cardiovascular diseases (Kobayashi et al., 2018), acute kidney failure (Müller et al., 2017) and

may also play a role in central degenerative brain disorders ((Weiland et al., 2019) (Yang and Stockwell, 2016). Ferroptosis is driven by loss of activity of lipid repair enzyme GPX4 and subsequent accumulation of lipid hydroperoxides. Depletion of GPX4 in mice is known to induce ferroptotic cell death in embryo, testis, brain, liver, heart, and photoreceptor cells (Imai et al., 2017), cause rapid motor neuron degeneration and paralysis (Chen et al., 2015), promotes cognitive impairment (Hambright et al., 2017), triggers acute renal failure (Friedmann Angeli et al., 2014), and results in impaired T-cell-mediated immune response (Matsushita et al., 2015). Mice with depleted GPX4 showed hallmarks of ferroptosis including an increase in lipid peroxidation in various cell types (Hambright et al., 2017).

There is no comparable therapy for SSMD to treat this condition, except for physical and occupational therapies. Antioxidants such as Vitamin E, N-Acetyl-Cysteine, Co Enzyme Q10 have been shown to inhibit ferroptosis in vitro. Ferrostatin-1 is known to reduce reactive oxygen species in vitro experiments. But they seldom show results across systems and few cross the blood brain barrier.

John Doe, although stable today, is at high risk for death by cardiovascular, cerebrovascular, neuromuscular, or renal complications. Severe hypotonia, abnormal bone development, and significantly delayed physical and cognitive development leads to substantial impact on day-to-day functioning. His skeletal findings and MRI show progression of the SSMD disease, but due to lack of documented data on natural history of the disease, his prognosis remains uncertain. Judging by the prognosis of other patients, evidence of ferroptosis leading to organ failures, and John's skeletal progression, we are concerned that John's survival is at risk.

EXAMPLE-001 is a strong drug known to tackle lipid peroxidation. Two of the normal phosphorus atoms have been replaced with hydrogen atoms observed lack of GPX4 activity leads to accumulation of PUFA hydroperoxides triggering ferroptosis, whereas pretreating cells with EXAMPLE-001 prevented the PUFA peroxidation thereby blocking ferroptosis.

John's loss-of-function variant is known to increase lipid peroxidation, whereas EXAMPLE-001 is effective at inhibiting the autocatalytic lipid peroxidation. By reducing the downstream effects of loss of GPX4 function, we believe EXAMPLE-001 has the potential to slow down or halt disease progression.

His condition, if left untreated, will progress to a more serious condition and affect not only his quality of life, but also his survival. The benefits of stopping or slowing down his disease progression outweighs the risks of administering an experimental drug. We believe EXAMPLE-001 could give John a chance at living a meaningful life he deserves.

3 Chemistry Manufacturing and Controls

All CMC information can be found in the Commercial Sponsor's IND submissions. There will be no change in the formulation, packaging, storage, or in dosing scheme (except as noted in Section 2.1.

4 Pharmacology and Toxicology Information

Pharmacology and Toxicology information is provided in the Commercial Sponsor's IND submissions.

5 Previous Human Experience

Previous human experience with EXAMPLE-001 is provided in the Commercial Sponsor's IND submissions. *Attachment 6 (Example Inc's Investigational Brochure and Rationale to Treat SSMD)* also provides detailed clinical data from several human studies.

6 References

Chen, L., Hambright, W.S., Na, R., Ran, Q., 2015. Ablation of the Ferroptosis Inhibitor Glutathione Peroxidase 4 in Neurons Results in Rapid Motor Neuron Degeneration and Paralysis. J. Biol. Chem. 290, 28097–28106. https://doi.org/10.1074/jbc.M115.680090

ClinVar Database https://www.ncbi.nlm.nih.gov/clinvar?term=GPX4%5Bgene%5D%20AND%20%22single%20gene%22%5BProperties%5D&cmd=DetailsSearch

Friedmann Angeli, J.P., Schneider, M., Proneth, B., Tyurina, Y.Y., Tyurin, V.A., Hammond, V.J., Herbach, N., Aichler, M., Walch, A., Eggenhofer, E., Basavarajappa, D., Rådmark, O., Kobayashi, S., Seibt, T., Beck, H., Neff, F., Esposito, I., Wanke, R., Förster, H., Yefremova, O., Heinrichmeyer, M., Bornkamm, G.W., Geissler, E.K., Thomas, S.B., Stockwell, B.R., O'Donnell, V.B., Kagan, V.E., Schick, J.A., Conrad, M., 2014. Inactivation of the ferroptosis regulator Gpx4 triggers acute renal failure in mice. Nat. Cell Biol. 16, 1180–1191. https://doi.org/10.1038/ncb3064

Hambright, W.S., Fonseca, R.S., Chen, L., Na, R., Ran, Q., 2017. Ablation of ferroptosis regulator glutathione peroxidase 4 in forebrain neurons promotes cognitive impairment and neurodegeneration. Redox Biol. 12, 8–17. https://doi.org/10.1016/j.redox.2017.01.021

Imai, H., Matsuoka, M., Kumagai, T., Sakamoto, T., Koumura, T., 2017. Lipid Peroxidation-Dependent Cell Death Regulated by GPx4 and Ferroptosis. Curr. Top. Microbiol. Immunol. 403, 143–170. https://doi.org/10.1007/82_2016_508

Kobayashi, M., Suhara, T., Baba, Y., Kawasaki, N.K., Higa, J.K., Matsui, T., 2018. Pathological Roles of Iron in Cardiovascular Disease. Curr. Drug Targets 19, 1068–1076. https://doi.org/10.2174/1389450119666180605112235

Matsushita, M., Freigang, S., Schneider, C., Conrad, M., Bornkamm, G.W., Kopf, M., 2015. T cell lipid peroxidation induces ferroptosis and prevents immunity to infection. J. Exp. Med. 212, 555–568. https://doi.org/10.1084/jem.20140857

Müller, T., Dewitz, C., Schmitz, J., Schröder, A.S., Bräsen, J.H., Stockwell, B.R., Murphy, J.M., Kunzendorf, U., Krautwald, S., 2017. Necroptosis and ferroptosis are alternative cell

death pathways that operate in acute kidney failure. Cell. Mol. Life Sci. CMLS 74, 3631–3645. https://doi.org/10.1007/s00018-017-2547-4

Smith, A.C., Mears, A.J., Bunker, R., Ahmed, A., MacKenzie, M., Schwartzentruber, J.A., Beaulieu, C.L., Ferretti, E., FORGE Canada Consortium, Majewski, J., Bulman, D.E., Celik, F.C., Boycott, K.M., Graham, G.E., 2014. Mutations in the enzyme glutathione peroxidase 4 cause Sedaghatian-type spondylometaphyseal dysplasia. J. Med. Genet. 51, 470–474. https://doi.org/10.1136/jmedgenet-2013-102218

Weiland, A., Wang, Y., Wu, W., Lan, X., Han, X., Li, Q., Wang, J., 2019. Ferroptosis and Its Role in Diverse Brain Diseases. Mol. Neurobiol. 56, 4880–4893. https://doi.org/10.1007/s12035-018-1403-3

Yang, W.S., Stockwell, B.R., 2016. Ferroptosis: Death by Lipid Peroxidation. Trends Cell Biol. 26, 165–176. https://doi.org/10.1016/j.tcb.2015.10.014

Attachment 1: Example Inc's Reference Rights Letter and Letter of Authorization

Attachment 2: John Doe – Medical History

Attachment 3: Clinical Presentation of other patients with p.R216H mutation

Attachment 4: Proposed Treatment Protocol

Attachment 5: Draft Informed Consent Form (ICF)

Attachment 6: Example Inc’s Investigational Brochure and Rationale to Treat SSMD

Attachment 7: CV – Dr. John Doe, MD, PhD
