# Supplementary material for: Development of therapies for rare genetic disorders of GPX4: roadmap and opportunities
Source: Orphanet J Rare Dis. 2021 Oct 23;16:446. doi: 10.1186/s13023-021-02048-0 (PMC8542321; doi:10.1186/s13023-021-02048-0)
Supplement: Supplementary file 2 — Additional file 2: Conference format and guide, [file 13023_2021_2048_MOESM2_ESM.pdf]

March 19, 2020

CURE GPX4 RESEARCH CONFERENCE

Together, we will find a cure...

CureGpx4.org

AGENDA, FORMAT & GUIDE

Thanks to our sponsor Salem Oaks

Attendees

Name	Institution
**Dr. Plavi Mittal**	Founder & CEO, In-Depth Genomics. Formerly Jain Foundation
**Dr. Ethan Perlstein**	CSO, Christopher & Dana Reeve Foundation
**Dr. Brent R. Stockwell**	Professor, Columbia University
**Dr. Simon Johnson**	Asst. Professor, University of Washington
**Dr. Qitao Ran**	Associate Professor, University of Texas at San Antonio
**Dr. Russel Saneto**	Neurologist, Seattle Children's Hospital
**Dr. Kristen Wigby**	Genetics Specialist, UC San Diego
**Dr. Reena Kartha**	Asst. Professor, University of Minnesota
**Dr. Lauren Black**	Distinguished Scientist, Charles River Laboratories
**Dr. Hugo Bellen**	Professor, Baylor College of Medicine
**Dorian M Cheff**	PhD Student, NCATS/Karolinska Institutet
**Dr. Elias Arner**	Professor, Karolinska Institute
**Dr. Matt Hall**	Biology Group Leader, NCATS
**Dr. Vamsi Mootha**	Professor, Harvard Medical School
**Dr. David Fisher**	Executive Director, Charles River

Name	Institution
**Dr. Andrew Crouse**	Director of Research, Hugh Kaul Precision Medicine Institute, University of Alabama Birmingham
**Sienna Rucka**	Student Analyst, Hugh Kaul Precision Medicine Institute, University of Alabama Birmingham
**Kevin Freiert**	Principal, Salem Oaks Consulting, Ex-Pfizer
**Dr. Thomas Trimarchi**	SVP, BridgeBio
**Dr. Tsz Leung To**	Staff Researcher, Harvard Medical School
**Dr. Eric Sid**	Program Officer, Office of Rare Disease Research, NCATS
**Dr. Lance Stewart**	Chief Strategy and Operations Officer, Institute of Protein Design, University of Washington
**Dr. Miguel Esteves**	Associate Professor, Univ of Massachusetts
**Dr. Mark Midei**	VP, Retrotope
**Dr. Vasanthi Viswanathan**	Researcher, Broad Institute
**Dr. John Aitchison**	Professor, Seattle Children's Research Institute
**Dr. Alysson Muotri**	Professor, Univ of California San Diego
**Dr. Matt Klein**	CEO, BioElectron
**Dr. Timothy Read**	CTO, Arpeggio Bio
**Dr. Qing Cheng**	Karolinska Institute

	Laboratories
**Hengrui Liu**	Columbia University

Agenda

All times are in pacific time zone (PST)

07:30 - 07:45 am Tech Check

Introduction & Keynote (08.00 to 09.00 am)

07.45 - 08.15 am Introductions and Welcome by *Sanath Kumar Ramesh*
08.15 - 08.45 am Keynote Speaker: Dr. David Fajgenbaum *"Chasing my cure"*
08.45 - 09.00 am Clinical Presentation of SSMD disease and Patient Needs from therapy by *Dr. Kristen Wigby*
09.00 - 9.30 am Break

Session 1: Basic Science (09.30 to 10.30 am)

09:30 - 09:35 am Explain the objective of the session by *Sanath Kumar Ramesh*
09.35 - 09.50 am "Context Talk" on GPX4 science by *Dr. Brent Stockwell*
09.50 - 10.30 am Workshop & Brainstorming: Basic Science moderated by *Dr. Brent Stockwell & Dr. Elias Arner*
10.30 - 10.45 am Break

Session 2: Small Molecule Drug Development (10.45 to 11.45 am)

10.45 - 10:50 am Explain the objective of the session by *Sanath Kumar Ramesh*
10.50 - 11.05 am "Context Talk" GPX4 Mouse Models by *Dr. Qitao Ran*
11.05 - 11.45 am Workshop & Brainstorming: Small Molecule Drug Development moderated by *Dr. Ethan Perlstein & Dr. Matt Hall*
11.45 - 12.15 pm Break

Session 3: Emerging Therapeutic Approaches (12.15 to 01.15 pm)

12.15 - 12:20 pm Explain the objective of the session by *Sanath Kumar Ramesh*
12.20 - 12:35 pm "Context Talk" on building gene therapy treatments by *Dr. Miguel Esteves*
12.35 - 01:15 pm Workshop & Brainstorming: Emerging therapeutic approaches moderated by *Dr. Plavi Mittal & Dr. Miguel Esteves*
01:15 - 01.45 pm Prioritize Roadmap & Closing Remarks

Conference Guide

Objective is to create a complete roadmap for therapy development by the end of the day. The roadmap will include identifying experiments necessary to understand disease, identifying small molecule drugs, and exploring the use of emerging technologies like gene therapy, ASOs etc to treat this condition. To accomplish this goal, we will have workshop-style free form discussions spread across three sessions.

Each session starts with a 10min "context" talk to get participants up-to-speed with the science underlying GPX4 with regard to its role in health and disease. This can provide the necessary background to all attendees to successfully participate and brainstorm. This will be followed by a ~40min brainstorming session. With the emphasis on informal freeform discussions, brainstorming sessions is where we expect everyone to contribute their expertise to help shape the roadmap.

The agenda, brainstorming session structure, and other activities are tailored to run a very collaborative online meeting respecting constraints such as multiple time zones, technology issues etc. We have dedicated time for tech-checks, ample breaks, dedicated note taking person, and a dedicated tech support person to ensure participants can focus on discussions.

Best Practices for Remote Conference

- Find a dedicated quiet room for the duration of the conference
- Use high-speed internet to support video conference of upto 20 people
- Turn on video to facilitate face-to-face interaction
- Mute yourself when you are not talking
- Recommended to use a headphone (no built-in speakers) to minimize echo
- Identify yourself before you speak in case people can’t see your video. A quick “Hey John Doe here..” before John Doe speaks will help everyone identify you
- Use the breaks as a good opportunity to interact with other participants on the online call

Roadmap Chart

See the next section for the Roadmap Chart which summarizes the current set of activities planned. Through the day, we will add more information to the chart as we learn in depth about GPX4. At the end of the day, we will summarize action items, prioritize activities and build a complete roadmap for the next year.

Session Structure

- Explain the objective (5mins)
- Context Talk (10mins + 5mins Q&A) to orient participants by covering enough topics necessary for brainstorming
- Brainstorm (40min)
    - Moderators will explain the objective of the session and start discussion with the first open ended question.
    - Discussions are freeform like a small "team meeting". Moderators' role is to guide discussion to meet the objective.
    - Participants can ask questions on chat or audio.
    - We will have a dedicated person to take notes during the session so everyone can follow along.
    - If new information is identified or a decision made, add it to "Roadmap Chart"

Roadmap Chart

Objective: Given 2yrs and $500 million dollars, find a therapy for GPX4 kids to provide a meaningful quality of life.

Strategy:

1. Repurpose approved drugs to slow down disease progression
2. De-risk the disease to make it attractive for industry partners
3. Develop new disease altering therapies to provide significant and meaningful improvements in quality of life

Disease	***Targets***	***Drug Candidates***	***Testing & Lead Identification***	***Commercialize***	***Clinical Trial / Compassionate Use***
**What do we know?**<br>(Dr. Wigby's talk)<br><br>- 10 Patients Worldwide<br>- SSMD Phenotype<br>- R152H is most common genotype	**What do we know?**<br>(Dr. Stockwell's talk)<br><br>GPX4<br>FSP1<br>NRF2<br>ACSL4	**What do we know?**<br>(Dr. Stockwell's talk)<br>Vitamin E<br>CoQ10<br>NAC<br>Selenium<br>Tecfidera<br>RT001<br>NACA<br><br>Gene therapy<br>(Dr. Esteves's talk)	**What do we know?**<br>(Dr. Ran's talk)<br><br>GPX4 KO mice characteristics	**What do we know?**	**What do we know?**<br>RT001 Compassionate use on Raghav
**What do we have?**	**What do we have?**<br>Recombinant mutant GPX4	**What do we have?**	**What do we have?**<br>- Conditional KO Mice<br>- Conditional KI Mice<br>- Patient-derived iPSCs<br>- Patient-derived Fibroblasts<br>- KO Fly	**What do we have?**	**What do we have?**
**What do we need?**<br><Brainstorm>	**What do we need?**<br><Brainstorm>	**What do we need?**<br><Brainstorm>	**What do we need?**<br><Brainstorm>	**What do we need?**<br><Brainstorm>	**What do we need?**<br><Brainstorm>

Session Guide for Presenters & Moderators

Introduction & Keynote	Session 1: Basic Science
**Dr. David Fajgenbaum "Chasing my cure" (30 min)**<br>"Chasing My Cure" is an amazing story of Dr.Fajgenbaum to find a treatment for Castleman Disease. It is a story of courage and perseverance even when the odds didn't work in his favor.<br><br>**Dr. Kristen Wigby's Talk (10mins + 5mins Q&A)**<br>- Diagnostic odyssey<br>- SSMD disease phenotype<br>- Genotypes<br>- MRIs, X-rays, other Clinical markers<br>- Life of patients	**Dr. Brent Stockwell's Talk (10mins + 5mins Q&A)**<br>- Role of GPX4 in health & disease<br>- R152H mutant data / biochemistry / recombinant protein<br>- Other genetic targets rescuing GPX4 loss<br>- GPX4 drug candidates<br>- Assays available today<br><br>**Workshop & Brainstorming**<br>**(Moderators: Dr. Brent Stockwell & Dr. Elias Arner)**<br>Objective: Identify next set of activities to understand the disease in order to facilitate therapy development<br><br>- Open questions about the disease?<br>- What experiments are necessary to understand disease (proteomics, metabolomics, lipidomics etc)?<br>- How do we identify disease biomarkers or surrogate markers?<br>- What do we know about similar diseases where high ROS is implicated?<br>- How can a natural history study help us understand disease?<br>- What are the biggest risks we should be worried about?

Session 2: Small Molecule Drug Development	Session 3: Emerging Technologies
**Dr. Qitao Ran's Talk (10mins + 5mins Q&A)**<br>- Types of mice available, characteristics of GPX4 loss in mice<br>- Drugs capable of rescuing mice<br>- Study to test 3 drugs (NAC+RT001, NAC+Tecfidera, NACA)<br><br>**Workshop & Brainstorming**<br>**(Moderators: Dr. Ethan Perlstein & Dr. Matt Hall)**<br>Objective: Identify next set of activities to repurpose existing drugs and de-risk disease to attract industry partners?<br><br>- Quick summary of models we are building<br>- How can we identify more approved drugs to repurpose?<br>- What pharmacological properties are important in drugs we consider?<br>- What is the value of discovering new (unapproved) molecules? How should we go about doing it?<br>- What endpoints/biomarkers/surrogate markers should we track in models and humans to quantify therapy outcome?<br>- What are the biggest risks we should be worried about?<br>- What should we do to de-risk the disease for industry to take on?<br>- How can computational modelling help identify potential therapies?<br>- How can we show business value by combining with related diseases (ex: Parkinsons, Cancer etc)?	**Dr. Miguel Esteves Talk (10mins + 5mins Q&A)**<br>- Gene therapy basics<br>- Challenges in neurological and skeletal diseases<br>- Cost breakdown<br>- GPX4 gene therapy high-level plan<br><br>**Workshop & Brainstorming**<br>**(Moderators: Dr. Plavi Mittal & Dr. Miguel Esteves)**<br>Objective: Explore gene therapy/ASO feasibility and identify next steps<br><br>- Is ASO for exon skipping a viable strategy?<br>- Can the Exon 6 be skipped to stay in the reading frame?<br>- Will protein function without exon 6?<br>- Tissue and organ systems to target with GPX4 gene therapy?<br>- Expression pattern of gene?<br>- Delivery and ideal AAV vectors?<br>- Defined measurable outcomes?<br>- Ideal mouse model suited for preclinical testing?<br>- Is it possible to perform gene therapy as an N-of-1 trial?<br>- Other diseases that benefit with GPX4 expression?<br>- Stage to de-risk for industry interest<br>- Other downstream genes to overexpress instead?<br>- How does CRISPR compare to gene therapy for GPX4?<br>- Pros vs Cons<br>- Timeline to Clinic?

Computer Setup for Online Conference

1. Install “Zoom Client For Meetings”: https://zoom.us/download#client_4meeting

2. Test your video and audio by joining the test meeting. Click this link to join the test meeting - https://zoom.us/test.

- Detailed guide on joining test meeting is here: https://support.zoom.us/hc/en-us/articles/115002262083
- Troubleshoot audio/video settings by using instructions here: https://support.zoom.us/hc/en-us/articles/201362623-Changing-settings-in-the-desktop-client-or-mobile-app

3. Familiarize with the Zoom Client interface by playing around in the test meeting:

- Mute Button
- Video enable/disable Button
- Chat Window
- Screen Share button

4. On the day of conference, join the Conference Bridge using the link sent via the calendar invite. Alternatively click here to join the conference bridge
