# Supplementary material for: Development of therapies for rare genetic disorders of GPX4: roadmap and opportunities
Source: Orphanet J Rare Dis. 2021 Oct 23;16:446. doi: 10.1186/s13023-021-02048-0 (PMC8542321; doi:10.1186/s13023-021-02048-0)
Supplement: Supplementary file 3 — Additional file 3: Roadmap chart template. [file 13023_2021_2048_MOESM3_ESM.pdf]

Roadmap Chart

Objective: Given 2yrs and $500 million dollars, find a therapy for GPX4 kids to provide a meaningful quality of life.

Strategy:

1. Repurpose approved drugs to slow down disease progression
2. De-risk the disease to make it attractive for industry partners
3. Develop new disease altering therapies to provide significant and meaningful improvements in quality of life

Disease	***Targets***	***Drug Candidates***	***Testing & Lead Identification***	***Commercialize***	***Clinical Trial / Compassionate Use***
**What do we know?**<br>(Dr. Wigby's talk)<br><br>- 10 Patients Worldwide<br>- SSMD Phenotype<br>- R152H is most common genotype	**What do we know?**<br>(Dr. Stockwell's talk)<br><br>GPX4<br>FSP1<br>NRF2	**What do we know?**<br>(Dr. Stockwell's talk)<br>Vitamin E<br>CoQ10<br>NAC<br>Selenium<br>Tecfidera<br>RT001<br>NACA<br><br><br>Gene therapy<br>(Dr. Esteves's talk)	**What do we know?**<br>(Dr. Ran's talk)<br><br>GPX4 KO mice characteristics	**What do we know?**<br><Brainstorm>	**What do we know?**<br>RT001 Compassionate use on Raghav
**What do we have?**<br><Brainstorm>	**What do we have?**<br>Recombinant mutant GPX4	**What do we have?**<br><Brainstorm>	**What do we have?**<br>- Conditional KO Mice<br>- Conditional KI Mice<br>- Patient-derived iPSCs<br>- Patient-derived Fibroblasts<br>- KO Fly	**What do we have?**<br><Brainstorm>	**What do we have?**<br><Brainstorm>
**What do we need?**<br><Brainstorm>	**What do we need?**<br><Brainstorm>	**What do we need?**<br><Brainstorm>	**What do we need?**<br><Brainstorm>	**What do we need?**<br><Brainstorm>	**What do we need?**<br><Brainstorm>
