# Supplementary material for: Development of therapies for rare genetic disorders of GPX4: roadmap and opportunities
Source: Orphanet J Rare Dis. 2021 Oct 23;16:446. doi: 10.1186/s13023-021-02048-0 (PMC8542321; doi:10.1186/s13023-021-02048-0)
Supplement: Supplementary file 4 — Additional file 4: Manual drug repurposing chart. [file 13023_2021_2048_MOESM4_ESM.pdf]

Low-Throughput Drug Repurposing

The following table contains a list of existing drugs (FDA approved or experimental) that could be effective. In SSMD disease, a mutation in GPX4 reduces the function of the protein. In a loss-of-function condition, our goal is to find drugs that:

1. Increase GPX4 protein levels and/or increase residual GPX4 activity
2. Increase the activity GPX4 antioxidant pathways by modifying the quantity of other participating proteins
3. Increase the activity of alternate compensatory pathways
4. Reduce or scavenge the phospholipid oxidation damage due to reduced GPX4 activity (e.g., use of antioxidants)
5. Drugs that have been found to be effective in similar conditions

Drugs are grouped into sections based on the mechanisms of actions. In each section, drugs are ordered by likelihood of therapeutic effect (high to low).

Increase GPX4 protein quantity (Category 1)

Rationale: Directly increase GPX4 protein quantity by histone modifications, changes to DNA methylation, activating GPX4 gene, or saturating the GPX4 production pathway.

**Name**	**FDA Status**	**Mechanism of Action**	**Ref**	**Decision**
Selenium	OTC	Providing more selenium will help saturate the GPX4 production pathways. But excess selenium is also harmful.		Yes
Nicotinamide	OTC	HDAC inhibitor to prevent deacetylation of histone which reduces transcription		NO<br><br>Ineffective

Increase pathway activity (Category 3)

Rationale: GPX4 catalyzes the antioxidant pathway along with several molecules like Glutathione, Glutathione Reductase, NADPH, Iron etc. We hypothesize that a partial loss of GPX4 function reduces the activity of this pathway. We identify drugs capable of compensating for the loss of activity by either increasing the pathway's activity or reducing the oxidant load.

**Name**	**FDA Status**	**Mechanism of Action**	**Ref**	**Decision**

N-Acetyl-Cysteine	OTC	Provides cysteine to increase the production of glutathione.		YES<br><br>Already administered
Methionine	OTC	Amino acid acting as a substrate to produce Cysteine. Could be used in combination with NAC.		NO<br><br>Ineffective. Risk of overload
Glutathione esters	OTC	Substitutes cellular pool of GSH. Known to work in-vivo.	(1)	NO<br><br>Not enough bioavailability
EPI743	Phase 3	Increase GSH synthesis. Has antioxidant property. But not available under expanded access		NO<br><br>Company does not do expanded access

Reduce generation of Reactive Oxygen Species (Category 2)

Rationale: Reduce the oxidant load in the cell to a point where the partial GPX4 activity is sufficient to maintain normal oxidative stress levels.

**Name**	**FDA Status**	**Mechanism of Action**	**Ref**	**Decision**
Zileuton	Rx	5-lipoxygenase inhibitor. Reduces ROS. Known to have antioxidant properties.	(2)	TBD<br><br>Need more understanding
BHT (Butylated Hydroxytoluene)	OTC	Food preservative. Known antioxidant. But chronic usage isn't recommended	(3)	NO<br><br>Mixed safety results
Apocynin	OTC	Inhibits NADPH Oxidase (Nox2) to control ROS production. Also an antioxidant.	(4, 5)	NO<br><br>No PKPD

				data. No drugs. But good science data
Deferoxamine	Rx	Iron chelator used in blood transfusion		NO<br><br>Side effects
Deferiprone	Rx	Iron chelator. Crosses blood-brain barrier, crosses cell membrane, redistributes inter-mitochondrial iron to extracellular network. Generally safe at low doses.	(6)	NO<br><br>Side effects

Increase activity of NRF-2 pathway (Category 3)

Rationale: NRF2 is a well-known Transcription Factor upregulated under high oxidative stress. (7) Several drugs are known to activate NRF2 (8) Due to the number of genes activated by NRF2, these drugs could give us better results by exploiting the body's natural defense mechanism. On the downside, it might be tricky to identify the right dose of drug because at very low or very high doses, NRF2 will not get activated.

**Name**	**FDA Status**	**Mechanism of Action**	**Ref**	**Decision**
Sulforaphane	OTC	Derived from broccoli. Shows 1.5-2x activation of NRF2 and downstream genes. Gold standard for NRF2 activation.	(9)	YES<br><br>Safe, but unsure
Tecfidera	Rx	Drug for multiple sclerosis. Better than Sulforaphane in NRF2 activation response.	(9)	YES<br><br>Safe. Effective.
Bardoxolone methyl	Phase 2	Better than Sulforaphane. Positive result in chronic kidney disease.	(1)	NO<br><br>Drug not available
RTA-408	Phase 3	Inhibits KEAP1 to induce NRF2		TBD

Baicalein	OTC	Herb found in Chinese medicine. Safe for humans, but the mechanism of action isn't completely understood. Believed to activate NRF-2	(10)	NO<br><br>Mixed results

Protect lipids from peroxidation (Category 4)

Rationale: Reactive oxygen species causes peroxidation of the lipid membrane leading to ferroptosis. Protecting the lipids will reduce cell degradation.

Name	FDA Status	Mechanism of Action	Ref	Decision
RT001	Phase 2	"Fireproofs" lipids from peroxidation by substituting a hydrogen to deuterium		YES<br><br>IND in progress
Omega-3 / DHA	OTC	Absorbed by lipid membranes. Could help repair oxidized lipids by saturating the repair pathway		NO<br><br>Interacts with RT001

Scavenge Reactive Oxygen Species (Category 4)

Rationale: Scavenge reactive oxygen species to reduce the oxidative stress enough to compensate for reduction in GPX4 activity.

Name	FDA Status	Mechanism of Action	Ref	Decision
Vitamin E	OTC	Well known antioxidant. Protects against lipid peroxidation		YES<br><br>Administered
Epicatechin	OTC	Natural flavanol derived from cacao plants. Antioxidant. Known to help skeletal muscle. Used in body building supplements.	(11)	YES<br><br>Crosses BBB. Safe.
Mitoquionone	OTC	CoQ10 analogue. Very well studied in literature. Enters mitochondria as antioxidant	(12)	YES

Ergothioneine	OTC	Naturally occurring amino acid acts as a weak antioxidant.<br>Importantly, it is known to synergistically amplify effect of NAC to protect cells	(13, 14)	NO<br><br>Weak results
Erdosteine	Rx	Mucolytic drug, but antioxidant. Very effective in COPD (smoking) and very safe.	(15)	TBD
Carbocysteine	Rx	Mucolytic drug, but antioxidant. Similar to NAC, but uses the thioester group.	(15)	TBD
Methylprednisolone (Lazaroids)	Rx	Highly effective to prevent lipid peroxidation after spinal cord injury. Used extensively in CNS. Steroid.	(16)	NO<br><br>Steroid side effects
VP-20629 / SH622	Phase 2	Derivative of naturally occurring putative antioxidant. Safe for humans.	(17)	NO<br><br>Drug unavailable
Edaravone	Rx	ALS Drug thought to be antioxidant. Intravenous administration.	(18)	NO<br><br>Intravenous
Folic acid	OTC	Known antioxidant. Naturally occurring		NO<br><br>Weak results

Prevent mitochondrial damage (Category 5)

Rationale: One isoform of GPX4 enters mitochondria. We hypothesize that excess ROS might affect mitochondria structure and function leading to phenotypes similar to classic mitochondrial conditions such as Leigh's syndrome.

Name	FDA Status	Mechanism of Action	Ref	Decision
MTP-131 aka Bendavia	Phase 3	Binds to cardiolipins found in the mitochondrial membrane to help the mitochondrial complex 1 to function more effectively.	(19)	YES<br><br>Worth trying

Leber's hereditary optic neuropathy (LHON) (Category 5)
Rationale: Increase in ROS might affect mitochondria structure and function.

Name	FDA Status	Mechanism of Action	Ref	Decision
Idebenone	Rx	CoQ10 derivative. But acts outside mitochondria. Very different PKPD from CoQ10. Antioxidant.	(20)	YES<br><br>Worth trying as CoQ10 alternative

High Blood Pressure / Heart Failure (Category 5)
Rationale: Several Beta-blockers have been shown to prevent lipid peroxidation. There are many known beta blockers (atenolol, labetalol, metoprolol, pindolol, propranolol, sotalol, timolol, and carvedilol), but here we have only those with good antioxidant properties.

Name	FDA Status	Mechanism of Action	Ref	Decision
Carvedilol	Rx	Beta Blocker. Reduces heart rate and blood pressure. Known to reduce oxidative stress and thereby heart failure.	(21)	NO<br><br>Causes fatigue
Propranolol	Rx	Similar to Carvedilol. Beta blocker but weak antioxidant	(22)	NO<br><br>Causes fatigue

Chemotherapy (Category 5)

Name	FDA Status	Mechanism of Action	Ref	Decision
Dexrazoxane	Rx	Used with chemotherapy drug to prevent cardiotoxicity. Known to reduce reactive oxygen species through a variety of mechanisms	(23)	TBD

Chronic Kidney Disease (Category 5)

Name	FDA Status	Mechanism of Action	Ref	Decision
Erythropoietin	Rx	Enzyme produced by the kidney responsible for RBC production. Known to decrease oxidative stress.	(24)	NO<br><br>Kidney side effects

Aging (Category 5)

Rationale: Oxidative stress is known to play a critical role in aging. It is interesting to examine the “miracle” drugs to find relevant ones.

Name	FDA Status	Mechanism of Action	Ref	Decision
Resveratrol	OTC	Occurs naturally in several plans, fruits, grapes etc. Very well known in the anti-aging community. Known to have antioxidant properties.		NO<br><br>Weak results

Cystic Fibrosis / Chronic Obstructive Pulmonary Disease (Category 5)

Many antioxidants have been tested with varying levels of success (25)

Spinal Cord Injury (Category 5)

Lipid peroxidation is a secondary effect of SCI. Antioxidants are used effectively. In-depth review with several drugs tested in models (26)

Friedreich’s Ataxia (Category 5)

Review drugs tried in FRDA (19)

References

1. Anderson ME, Luo JL. Glutathione therapy: from prodrugs to genes. Semin Liver Dis. 1998;18(4):415-24.

2. Saul D, Gleitz S, Nguyen HH, Kosinsky RL, Sehmisch S, Hoffmann DB, et al. Effect of the lipoxygenase-inhibitors baicalein and zileuton on the vertebra in ovariectomized rats. Bone. 2017;101:134-44.
3. Yehye WA, Rahman NA, Ariffin A, Abd Hamid SB, Alhadi AA, Kadir FA, et al. Understanding the chemistry behind the antioxidant activities of butylated hydroxytoluene (BHT): a review. Eur J Med Chem. 2015;101:295-312.
4. Sovari AA, Morita N, Karagueuzian HS. Apocynin: a potent NADPH oxidase inhibitor for the management of atrial fibrillation. Redox Rep. 2008;13(6):242-5.
5. t Hart BA, Copray S, Philippens I. Apocynin, a low molecular oral treatment for neurodegenerative disease. Biomed Res Int. 2014;2014:298020.
6. Pandolfo M, Arpa J, Delatycki MB, Le Quan Sang KH, Mariotti C, Munnich A, et al. Deferiprone in Friedreich ataxia: a 6-month randomized controlled trial. Ann Neurol. 2014;76(4):509-21.
7. Holmström KM, Kostov RV, Dinkova-Kostova AT. The multifaceted role of Nrf2 in mitochondrial function. Curr Opin Toxicol. 2016;1:80-91.
8. Hybertson BM, Gao B, Bose SK, McCord JM. Oxidative stress in health and disease: the therapeutic potential of Nrf2 activation. Mol Aspects Med. 2011;32(4-6):234-46.
9. Petrillo S, Piermarini E, Pastore A, Vasco G, Schirinzi T, Carrozzo R, et al. Nrf2-Inducers Counteract Neurodegeneration in Frataxin-Silenced Motor Neurons: Disclosing New Therapeutic Targets for Friedreich's Ataxia. Int J Mol Sci. 2017;18(10).
10. Qin S, Deng F, Wu W, Jiang L, Yamashiro T, Yano S, et al. Baicalein modulates Nrf2/Keap1 system in both Keap1-dependent and Keap1-independent mechanisms. Arch Biochem Biophys. 2014;559:53-61.
11. Shay J, Elbaz HA, Lee I, Zielske SP, Malek MH, Hüttemann M. Molecular Mechanisms and Therapeutic Effects of (-)-Epicatechin and Other Polyphenols in Cancer, Inflammation, Diabetes, and Neurodegeneration. Oxid Med Cell Longev. 2015;2015:181260.
12. Smith RA, Murphy MP. Animal and human studies with the mitochondria-targeted antioxidant MitoQ. Ann N Y Acad Sci. 2010;1201:96-103.
13. Aruoma OI, Spencer JP, Mahmood N. Protection against oxidative damage and cell death by the natural antioxidant ergothioneine. Food Chem Toxicol. 1999;37(11):1043-53.
14. Cheah IK, Halliwell B. Ergothioneine; antioxidant potential, physiological function and role in disease. Biochim Biophys Acta. 2012;1822(5):784-93.
15. Rogliani P, Matera MG, Page C, Puxeddu E, Cazzola M, Calzetta L. Efficacy and safety profile of mucolytic/antioxidant agents in chronic obstructive pulmonary disease: a comparative analysis across erdosteine, carbocysteine, and N-acetylcysteine. Respiratory Research. 2019;20(1):104.
16. Kavanagh RJ, Kam PCA. Lazaroids: efficacy and mechanism of action of the 21-aminosteroids in neuroprotection. BJA: British Journal of Anaesthesia. 2001;86(1):110-9.
17. Pharmaceutical S. NCT01898884: Safety and Pharmacology Study of VP 20629 in Adults With Friedreich's Ataxia: U.S. National Library of Medicine; 2020 [updated 05 Dec 2018. Available from: https://www.clinicaltrials.gov/ct2/show/NCT01898884.
18. Cruz MP. Edaravone (Radicava): A Novel Neuroprotective Agent for the Treatment of Amyotrophic Lateral Sclerosis. P T. 2018;43(1):25-8.

19. Clay A, Hearle P, Schadt K, Lynch DR. New developments in pharmacotherapy for Friedreich ataxia. Expert Opinion on Pharmacotherapy. 2019;20(15):1855-67.
20. Gueven N, Woolley K, Smith J. Border between natural product and drug: comparison of the related benzoquinones idebenone and coenzyme Q10. Redox biology. 2015;4:289-95.
21. Nakamura K, Kusano K, Nakamura Y, Kakishita M, Ohta K, Nagase S, et al. Carvedilol decreases elevated oxidative stress in human failing myocardium. Circulation. 2002;105(24):2867-71.
22. Gomes A, Costa D, Lima JL, Fernandes E. Antioxidant activity of beta-blockers: an effect mediated by scavenging reactive oxygen and nitrogen species? Bioorg Med Chem. 2006;14(13):4568-77.
23. Stěrba M, Popelová O, Vávrová A, Jirkovský E, Kovaříková P, Geršl V, et al. Oxidative stress, redox signaling, and metal chelation in anthracycline cardiotoxicity and pharmacological cardioprotection. Antioxid Redox Signal. 2013;18(8):899-929.
24. Adamowicz A, Trafikowska U, Trafikowska A, Zachara B, Manitius J. Effect of erythropoietin therapy and selenium supplementation on selected antioxidant parameters in blood of uremic patients on long-term hemodialysis. Med Sci Monit. 2002;8(3):Cr202-5.
25. Domej W, Oettl K, Renner W. Oxidative stress and free radicals in COPD--implications and relevance for treatment. Int J Chron Obstruct Pulmon Dis. 2014;9:1207-24.
26. Hall ED. Antioxidant Therapies for Acute Spinal Cord Injury. Neurotherapeutics. 2011;8(2):152-67.
