# Supplementary material for: Development of therapies for rare genetic disorders of GPX4: roadmap and opportunities
Source: Orphanet J Rare Dis. 2021 Oct 23;16:446. doi: 10.1186/s13023-021-02048-0 (PMC8542321; doi:10.1186/s13023-021-02048-0)
Supplement: Supplementary file 5 — Additional file 5: Weekly status reports template [file 13023_2021_2048_MOESM5_ESM.pdf]

CUREGPX4 SCIENCE TEAM STATUS REPORT
15-June-2020

GOAL: Find a treatment to slow down disease progression by end of 2020

HIGHLIGHTS SINCE LAST REPORT

- We are waiting on one final paperwork to be signed before starting on experimental therapy.
- We have started producing iPSCs from fibroblasts for both patient and control lines.
- We are working on starting a natural history program.

SUMMARY
We are focusing on small molecule drug repurposing to achieve the goal. We have started patients on a few existing FDA approved drugs (Vit-E, N-Acetylcysteine, CoQ10, Selenium) and submitted the FDA application to try an experimental therapy. We will test more drugs as part of a pipeline on several GPX4 disease models to identify more efficacious drugs. We are exploring gene therapy as a long-term treatment.

AT A GLANCE
Number of GPX4 patients worldwide: 9
Team: https://www.curegpx4.org/team
Roadmap: https://www.curegpx4.org/roadmap
Disease models:

- Mouse: Conditional GPX4 Knockout (JAX, Stock No: 027964)
- Fibroblasts: Patient and Parent Control lines (RUCDR Biorepository)
- iPSCs: Patient and Parent Control lines (RUCDR Biorepository)
- Mouse: Conditional Knock-in R152H mutation (ETA: 1-Feb-2021)
- Fly: Knock-out or Knock-in (depending on viability of phenotype)

ACTIVE PROJECTS

**Project**	**Investigator**	**Status**	**Updates**
Natural History Study	Dr. John Doe	Preparation	We have a call set up an epidemiologist to finalize next steps for natural history study.
Gene Therapy	Dr. John Doe	Started	We have started building the gene therapy treatment

UPCOMING PROJECTS

**Project**	**Investigator**	**Status**	**Updates**

LINKS

Links to documents relevant to your work
